# Absolute Quantification of Viable but Nonculturable Vibrio cholerae Using Droplet Digital PCR with Oil-Enveloped Bacterial Cells

**DOI:** 10.1128/spectrum.00704-22

**Published:** 2022-06-28

**Authors:** Shuo Zhao, Xin Lu, JingYun Zhang, Biao Kan

**Affiliations:** a State Key Laboratory of Infectious Disease Prevention and Control, National Institute for Communicable Disease Control and Prevention, Chinese Center for Disease Control and Prevention, Beijing, China; University of Maryland Eastern Shore

**Keywords:** viable but noncultivable state, *Vibrio cholerae*, aquatic environment, ddPCR, propidium monoazide, qPCR, viable cell counting

## Abstract

When exposed to adverse conditions, many bacterial pathogens, including Vibrio cholerae, can adapt to the environment by entering the viable but nonculturable (VBNC) state. Cells in this state cannot grow on conventional media but still survive. The VBNC state is a significant threat to aquatic safety and public health due to the enhanced ability of the bacteria to remain in the environment and escape from monitoring. Detecting and quantifying VBNC cells and distinguishing them from nonviable cells is necessary for microbiological studies and pathogen monitoring. Cell staining and microscopy are used for the observation of VBNC cells, but it is difficult to quantify VBNC cells accurately. In this study, we developed droplet digital PCR (ddPCR) with a chromosomal single-copy gene as an internal reference combined with Propidium monoazide (PMA) treatment to enumerate VBNC cells of V. cholerae. In this method, bacterial cells, but not extracted chromosomal DNA, were used directly to form oil-enveloped droplets in the ddPCR procedure. One bacterial cell possesses one copy of the chromosome. Thus, enumeration of a single-copy gene on the chromosome can be used to count VBNC cells. ddPCR showed greater accuracy and sensitivity than qPCR. This study showed that the oil-enveloped bacterial method can reduce the number of steps needed and improve the accuracy of VBNC cells quantification and has the potential to be extended to quantify bacterial VBNC cells and assess pathogen survival in the environment.

**IMPORTANCE** The viable but nonculturable (VBNC) state of bacteria represents an important life state for their survival in adverse environments. The VBNC cells of the pathogenic bacteria in the environment and food will be a potential threat to public health because these pathogens cannot be found by the detection of culture. We developed a sensitive molecular method to detect and enumerate the VBNC cells of V. cholerae, which can distinguish the VBNC and dead cells, and count the VBNC cells in the sample without the step of DNA extraction from cells. It can be used to improve the sensitivity of pathogen detection in the surveillance, risk assessment of environment and food contamination, and outbreak warning. The accurate identification and numeration of VBNC cells will also facilitate the microbiological and genetic studies on the development, adaptation, resuscitation, and elimination of the VBNC state.

## INTRODUCTION

Bacteria entering the viable but noncultivable (VBNC) state are in a unique physiological state, and this behavior represents an important survival strategy for adaptation to adverse environments. Especially for pathogenic bacteria, the VBNC state can help to resist fatal or inhibitive factors in the environment and food and generate potential health threats. Vibrio cholerae in the aquatic environment is transmitted orally through contaminated water (1). In some cases, V. cholerae may be difficult to isolate from water due to conditions that are not conducive to proliferation (i.e., starvation, low-temperature irradiation, and antibiotic pressure) (2–5). Such bacteria can enter a dormant state, namely, the viable but nonculturable (VBNC) state (6). Bacteria in the VBNC state cannot grow on commonly used culture media but are still alive and can regain culturability when exposed to favorable conditions (7).

Considering the difficulty of isolating VBNC cells with the commonly used culture methods and the significance of VBNC cells for public health, the development of a detection strategy is particularly important (8). Direct microscopic counting of live marine bacteria is applied for early detection (9), but the unculturable nature of VBNC cells leads to low detection. The fluorescent redox probe CTC (5-cyano-2,3-ditolyl-tetrazolium chloride) was developed for detecting VBNC cells, but a high CTC concentration killed the cells, and the number of viable cells was greatly underestimated (10). Later, the live/dead stain BacLight was used to assess VBNC cell viability (11). Other staining molecules have been applied to trace VBNC cells, such as two fluorescent stains based on cytoplasmic membrane integrity, SYTO 9, and propidium iodide PI, combined with microscopic observation or flow cytometry (12). Propidium monoazide (PMA) can penetrate damaged cell membranes, which inhibits its amplification, for the enumeration of VBNC cells (13). PMA combined with quantitative real-time PCR (PMA-qPCR) or real-time LAMP (PMA-qLAMP) improves the accuracy of VBNC cell quantification (14–16), but this method relies on standard curve quantification and amplification efficiency.

Droplet digital PCR (ddPCR) can quantify and directly count the number of DNA molecules in samples. This approach does not require a standard curve and is not affected by amplification efficiency. ddPCR showed good consistency with qPCR in bacterial cell counting (17, 18) and was even more sensitive than qPCR in the counting of VBNC cells (19). Combined with PMA treatment, ddPCR can quantify the survival rate of bacteria (18, 19).

Previously used PMA-ddPCR assays require chromosomal DNA extraction of the sample, which is time-consuming. ddPCR starting with nanodroplets containing single bacterial cells has been tested and applied in the detection of bacteria and their target genes (20–22). In this study, we developed a method based on oil-enveloped bacterial cells (without DNA extraction) for ddPCR combined with PMA treatment to improve the rapid and absolute quantification of VBNC cells of V. cholerae. The study showed that oil-enveloped bacterial droplet digital PCR of single-copy genes combined with PMA treatment is a rapid and effective approach for counting VBNC cells in samples.

## RESULTS

### Sensitivity of qPCR and ddPCR quantification.

We determined the sensitivity of qPCR and ddPCR assays for V. cholerae traditional DNA extraction and direct oil-enveloped bacterial methods. The mean concentration of V. cholerae strain C6706 cells was approximately 6.3 × 10^8^ CFU/mL, which was determined by plate counting. Chromosomal DNA extracted from V. cholerae strain C6706 was used as the template for amplification at 13 ng/μL to 13 fg/μL.

Traditional DNA extraction methods use metal baths and DNA extraction kit methods. The slope of the quantitative standard curve was determined by qPCR, and the slopes were −3.68 and −3.35 for the metal bath and DNA kit, respectively. The average qPCR efficiencies of the metal bath and DNA kits were 87% and 99%, respectively. The lower limit of detection using SYBR green (tested in triplicate) was 13 fg/μL of total DNA used in the test, which means that the Cq value was 35.4 ± 0.95 and 33.78 ± 0.3 for the metal bath and DNA kit, respectively (Table 1). The lower limit of detection of DNA copies/μL for qPCR was 14.47 and 7.83 for the metal bath and DNA kit, respectively. For ddPCR, the lower limit (13 fg/μL) quantification was 3.6 and 3.3 copies/μL for the metal bath and DNA kit, respectively.

**TABLE 1:** Results from three quantification methods (oil-enveloped bacteria, metal bath, and DNA kit) involving qPCR and ddPCR from a 10-fold serial dilution of V. cholerae strain C6706

Concentration of template	Oil-enveloped bacteria		Metal bath		DNA kit	
	Cq mean or Copies/μL	SD	Cq mean or Copies/μL	SD	Cq mean or Copies/μL	SD
qPCR						
13 ng/μL	12.16	0.06	13.42	0.07	13.89	0.02
1.3 ng/μL	15.29	0.2	16.84	0.14	17.40	0.06
130 pg/μL	18.91	0.05	21.62	0.32	20.86	0.15
13 pg/μL	22.48	0.76	25.78	0.53	24.63	0.34
1.3 pg/μL	26.05	0.52	29.28	0.36	27.83	0.6
130 fg/μL	29.37	0.16	31.67	0.37	30.98	0.37
13 fg/μL	33.63	0.25	35.4	0.95	33.78	0.3
R^2^	0.999		0.992		0.998	
ddPCR						
13 ng/μL	ULOD^*a*^	ULOD	ULOD	ULOD	ULOD	ULOD
1.3 ng/μL	186000	7071.07	178000	5656.85	161900	8626.7
130 pg/μL	16900	2927.42	12050	2305.17	10730	1258.65
13 pg/μL	1240	466.69	1062	308.3	1450	523.26
1.3 pg/μL	186	49.50	86	5.66	180.5	6.36
130 fg/μL	24	9.89	18.1	3.54	25	9.9
13 fg/μL	3.5	2.40	3.6	1.70	3.3	1.56
R^2^	0.996		0.992		0.999	

a ULOD, DNA concentration at which the signal of the assay was saturated (>20,000 copies in the reaction mixture).

We attempted to optimize the establishment of a rapid detection method for oil-enveloped bacteria. This method requires (i) mixing the gradient dilution of the bacterial solution with the Premix for qPCR and the EvaGreen Supermix for ddPCR into a 20 μL system; (ii) DNA was released and lysed through qPCR and ddPCR cycle amplification; (iii) cycling at 95°C, followed by cold incubation at 37°C 10 min later, 10 min for qPCR (qPCR minimum temperature 37°C), and 4°C for 10 min for ddPCR. The results showed that cold incubation did not affect the qPCR and ddPCR results. The slope of the standard curve constructed by qPCR was −3.56. The amplification efficiency was 91%. The cq value of 13 fg/μL was 33.63 ± 0.25, which corresponds to the lower limit of detection for qPCR results of 13.90 copies/μL. The lower limit of quantification (13 fg/μL) of ddPCR was 3.5 copies/μL. The two PCR methods showed a good linear relationship in the quantitative range, and the *R*^2^ value was between 0.992 and 0.999.

The correspondence among CFU counting, PMA-qPCR, and PMA-ddPCR was analyzed, three methods had the same trend of change. Given the different counting methods, counting targets (colonies and chromosomal DNA), and influence factors, differences in absolute values were observed (Fig. S1).

### Enumeration of VBNC cells with qPCR and ddPCR on day 40.

**FIG 1:**
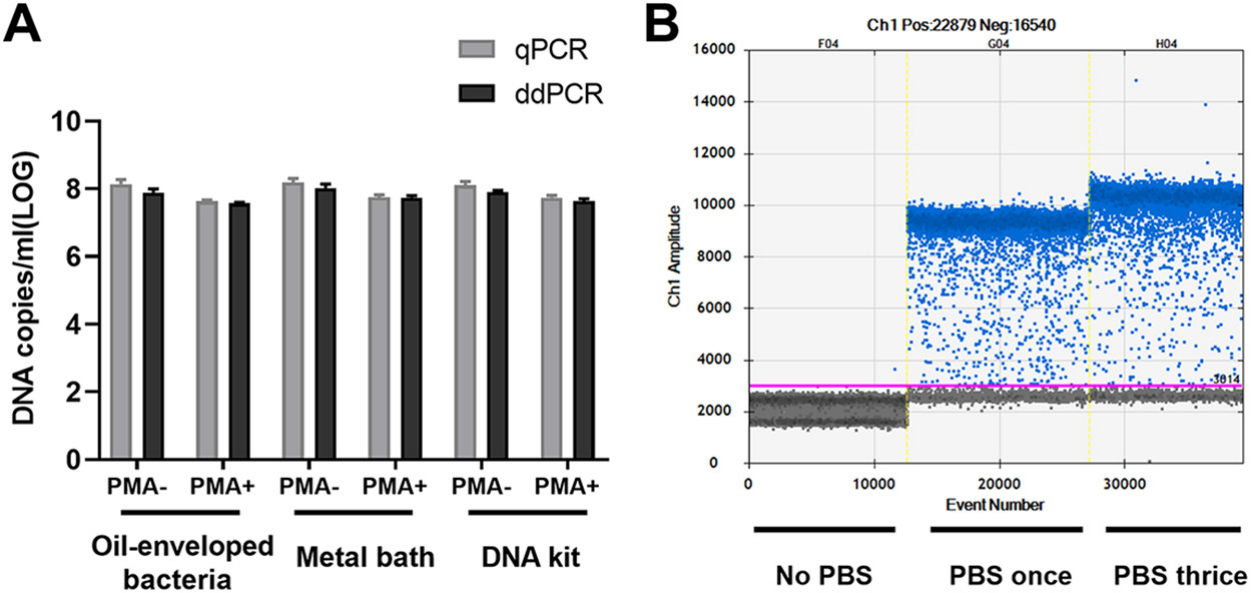
Quantification of total and viable cells (in DNA copies/mL) using oil-enveloped bacteria, a metal bath, and a DNA kit on day 40 during viable but nonculturable (VBNC)-state development of Vibrio cholerae strain C6706. (A) Total cells were measured without PMA treatment (PMA^−^), and viable cells were measured after PMA treatment (PMA^+^). qPCR, quantitative PCR; ddPCR, droplet digital PCR. Error bars represent the mean and standard error of measurement. (B) The samples were treated with PMA, and the oil-enveloped bacteria method was used to obtain ddPCR results without PBS washing and with PBS washing one and three times.

The samples were collected on day 40 of V. cholerae VBNC state development. Both qPCR and ddPCR were used to quantify DNA copies/mL from 1 × 10^8^ CFU/mL VBNC cells suspension. All three methods (oil-enveloped bacteria, metal bath, and DNA kit) were used as targets for enumeration. Total VBNC cells and VBNC-state living cells were enumerated by PCR without or with PMA treatment (Fig. 1A). When samples were treated with PMA, if the oil-enveloped bacteria method did not include a wash with PBS, ddPCR could not analyze the results according to the Poisson distribution. Interestingly, after a PBS wash, ddPCR showed good differentiation of negative and positive droplets. There was no significant difference in digital PCR results between washing with PBS once and thrice (Fig. 1B). However, the qPCR results were not affected by the inclusion or omission of a PBS wash.

Similar numbers of total cells (i.e., live + dead) were obtained by ddPCR using an oil-enveloped bacteria and DNA kit (7.89 ± 0.19 and 7.93 ± 0.15 log_10_ DNA copies/mL, respectively), and slightly higher values were obtained using a metal bath (8.02 ± 0.2 log_10_ DNA copies/mL). Quantification of viable cells by ddPCR gave values of 7.55 ± 0.1 and 7.57 ± 0.13 log_10_ DNA copies/mL using oil-enveloped bacteria and DNA kits, respectively. A slightly higher value was determined using a metal bath (7.64 ± 0.12 log_10_ DNA copies/mL).

The loss of cellular viability was evaluated from the cell counts determined by qPCR or ddPCR and the counts determined by PMA-qPCR or PMA-ddPCR, respectively. Using qPCR, the viability loss determined using oil-enveloped bacteria, the metal bath, and DNA kit was 0.53, 0.50, and 0.44 log_10_ DNA copies/mL, respectively. Using ddPCR, the viability loss determined using oil-enveloped bacteria, the metal bath, and DNA kit was 0.34, 0.38, and 0.36 log_10_ DNA copies/mL, respectively.

The consistency of the ddPCR-derived data was better than that of data obtained by qPCR when tested with samples from day 40 of VBNC-state development (Table 2). Here, we calculated the proportion of VBNC-state cells using the absolute value of live cells (DNA copies/mL in samples with PMA treatment) and the total number of DNA copies/mL (samples without PMA treatment). Quantification of the VBNC cell proportion by qPCR was 29.74%, 31.62%, and 36.31% when determined using oil-enveloped bacteria, a metal bath, and a DNA kit, respectively. However, when measured using ddPCR, the VBNC cell proportions were 46.06%, 41.69%, and 43.65% for oil-enveloped bacteria, the metal bath, and the DNA kit, respectively.

**TABLE 2:** Assessment of the viability loss rate of V. cholerae strain C6706 on day 40 of VBNC-state development by quantification using oil-enveloped bacteria, a metal bath, and a DNA kit for qPCR and ddPCR

Gene	Method	VBNC rate	Viability loss rate
Oil-enveloped bacteria	qPCR	29.74%	70.26%
	ddPCR	46.06%	53.94%
Metal bath	qPCR	31.62%	68.38%
	ddPCR	41.69%	58.31%
DNA kit	qPCR	36.31%	63.69%
	ddPCR	43.65%	56.35%

### Quantification of viable cells during VBNC-state development.

**FIG 2:**
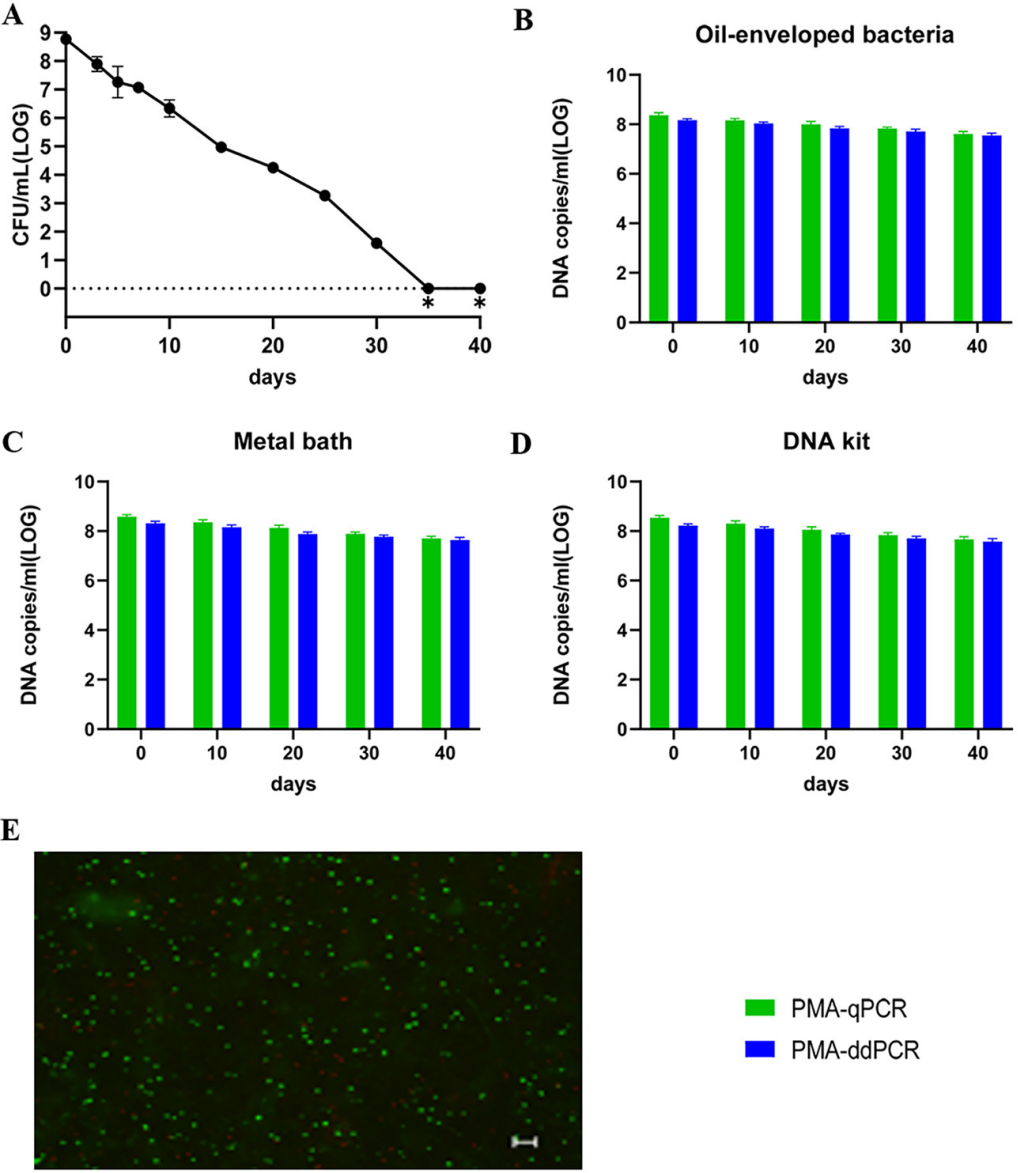
Quantification of viable cells by qPCR and ddPCR during VBNC state development of V. cholerae strain C6706. (A) Culturable cell counts during VBNC state development of strain C6706. ∗ indicates that the culturable cell count was <1 CFU/mL. (B to D) DNA copies/mL were measured using oil-enveloped bacteria, a metal bath, and a DNA kit after PMA treatment. (E) Live/dead staining of cells of V. cholerae strain C6706 on day 40 of VBNC state development (scale bar = 20 μm). Viable cells, which are stained only by SYTO9, appear green; dead bacteria appear red.

Development of the VBNC state of V. cholerae is a process. Day 40 was the endpoint of observation of the VBNC state in our study. Culture samples with and without PMA treatment were collected on days 0, 10, 20, 30, and 40. Three methods (oil-enveloped bacteria, metal bath, and DNA kit) were used to release DNA, followed by qPCR and ddPCR. The culturable cell counts (determined on LB agar plates) decreased gradually and fell to zero after 35 days (Fig. 2A). Viable cell counts were confirmed by live/dead staining, PMA-qPCR, and PMA-ddPCR (Fig. 2). The viable cell counts determined by PMA-qPCR and PMA-ddPCR using oil-enveloped bacteria, a metal bath, and a DNA kit showed a slow decrease from day 0 to day 40 (Fig. 2B to D). Based on the data presented in Fig. 2B to D, a slightly lower value of DNA copies/mL was obtained by PMA-ddPCR than by PMA-qPCR at each time point, and the values obtained by qPCR had a higher variance than the ddPCR data. Therefore, ddPCR had higher repeatability than qPCR for the three counting methods.

### Quantification of V. cholerae VBNC cells in mock aquatic samples.

**FIG 3:**
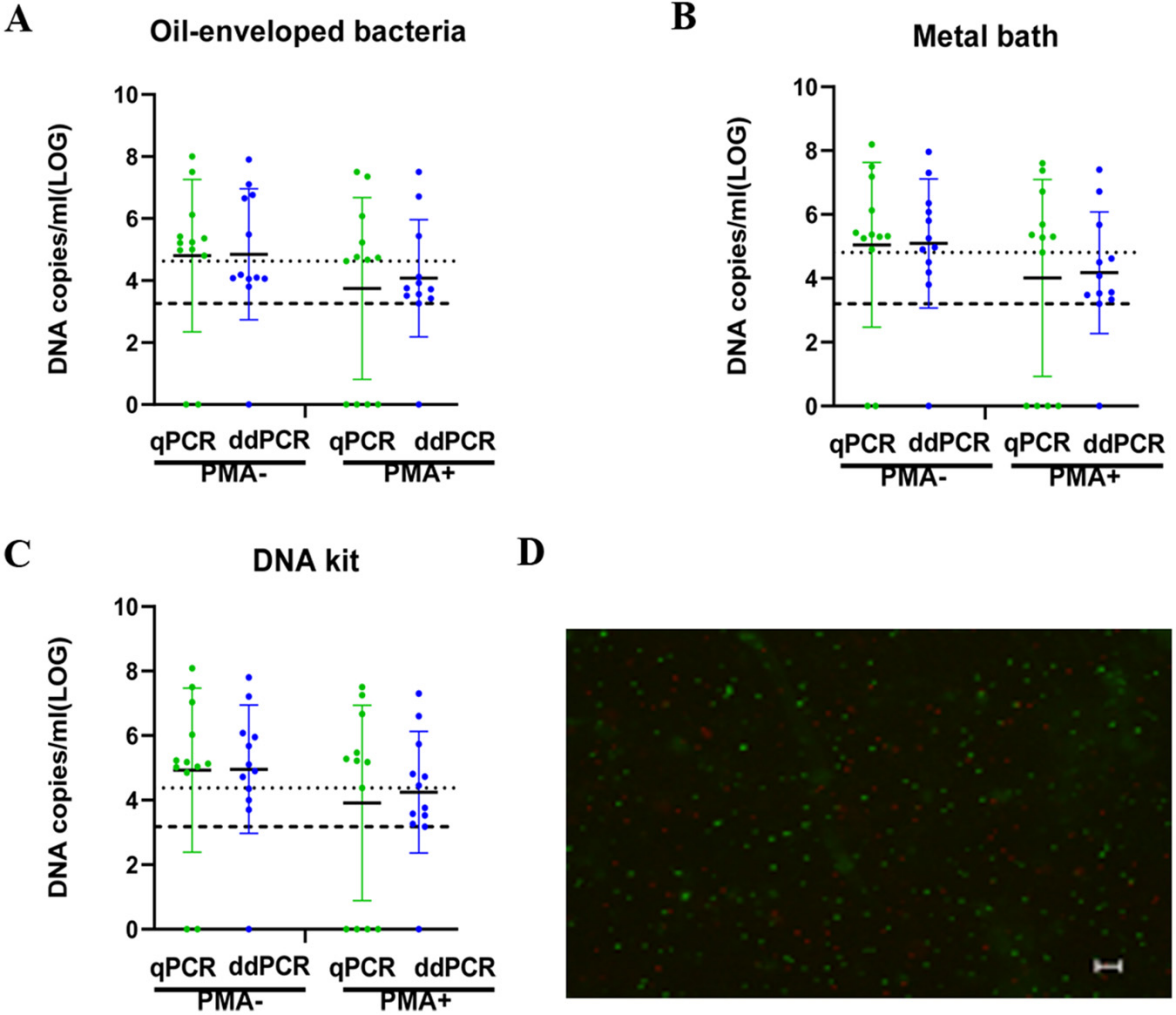
Quantification of total and viable cells (in DNA copies/mL) using oil-enveloped bacteria, a metal bath, and a DNA kit to analyze the V. cholerae VBNC state in an aquatic environment. (A to C) DNA copies/mL were measured using oil-enveloped bacteria, a metal bath, and a DNA kit to analyze total and viable cells. Total cell counts were obtained without PMA treatment, and viable cell counts were obtained after PMA treatment. The dotted line represents the qPCR lower limit of detection, and the dashed line represents the ddPCR lower limit of detection. (D) Live/dead staining of cells in the V. cholerae VBNC state in an aquatic environment (scale bar = 20 μm). Viable cells, which are stained only with SYTO9, appear green; dead bacteria appear red.

To test the applicability of qPCR and ddPCR with oil-enveloped bacteria for VBNC cell quantification in environmental water samples. V. cholerae VBNC state cells at day 40 were diluted, and each dilution was added to pond water samples (1:1, V/V). When total V. cholerae cells were measured in PMA-untreated samples, both the qPCR and ddPCR data showed no significant difference and confirmed that there was good adequacy between the two methods (Fig. 3). The quantitative values for the total number of cells (live + dead) in PMA-untreated samples by qPCR were 4.8 ± 2.46, 5.04 ± 2.58, and 4.92 ± 2.54 log_10_ DNA copies/mL for oil-enveloped bacteria, the metal bath, and the DNA kit, respectively. Similar numbers of total cells quantified by ddPCR were 4.85 ± 2.11, 5.09 ± 2.02, and 4.95 ± 1.99 log_10_ DNA copies/mL for oil-enveloped bacteria, the metal bath, and the DNA kit, respectively.

For PMA-treated samples quantified by qPCR in 8/12 samples, as expected, the number of live bacteria was slightly lower than the total number and very close to the qPCR lower limit of detection. However, 11/12 samples were quantified by ddPCR. Accuracy was greatly improved, and the lower limit of detection was reduced. The mean values of qPCR and ddPCR were 3.91, 4.09, and 4.08 log_10_ DNA copies/mL for oil-enveloped bacteria, the metal bath, and the DNA kit, respectively. The lower limit of the ddPCR value (1.37, 1.61, and 1.2 log_10_ DNA copies/mL for oil-enveloped bacteria, metal bath, and DNA kit, respectively) was higher than that of qPCR, confirming the results described in the second paragraph.

qPCR or ddPCR analysis was performed without PMA treatment to quantify the total number of cells in mock aquatic samples, and qPCR or ddPCR combined with PMA treatment was used to quantify the number of live VBNC cells. The loss of VBNC viability was evaluated by the difference between the PMA-qPCR or PMA-ddPCR value and the qPCR or ddPCR value. For qPCR and ddPCR, the mean values for the loss of VBNC viability were 0.91, 0.98, and 0.86 log_10_ DNA copies/mL for oil-enveloped bacteria, the metal bath, and the DNA kit, respectively.

## DISCUSSION

Culturable cell counts and the distinction between viable and dead cells in the VBNC state are strain-specific quantitative indicators that reflect VBNC state development. For the 40th day of V. cholerae VBNC culture, the conventional culture method could not form colonies, but live/dead staining shows that green fluorescence represents living cells. The differentiation of VBNC-state V. cholerae from dead cells is challenging, especially in aquatic environmental samples (8). In this study, we developed a method for the quantification of VBNC cells of V. cholerae by using PMA-ddPCR and starting from oil-enveloped bacteria, demonstrating rapid and accurate quantification with this protocol.

One of the major differences between qPCR and ddPCR quantification was the detection limit of the target copy number. The lower limit of detection of qPCR in 13 fg/μL of total DNA was 7.83 to 14.47 DNA copies/μL, while the lower limit quantification of ddPCR was 3.3 to 3.6 DNA copies/μL. In an aquatic environment, VBNC sample quantification, the lower limit of detection of ddPCR was 2.48 to 2.88 log_10_ DNA copies/mL, which was higher than that of qPCR. This difference was particularly important, especially because ddPCR can be used to quantify accurate results when there is a small number of VBNC cells. Therefore, ddPCR was used to quantify VBNC cells in more samples (i.e., water, food, clinical and drug-resistant bacteria). It has previously been reported that qPCR cannot accurately detect a small number of VBNC cells (23). Compared with qPCR, ddPCR has a greater ability to quantify a small number of target molecules, which is also consistent with data in the literature (18, 19).

In this study, we selected three different methods to quantify VBNC cells. One purpose was to use these methods technology for parallel verification of the quantitative method; another was to compare the results of the different DNA extraction methods. The oil-enveloped bacteria method was rapid and accurate for quantification of the VBNC total bacterial count (without PMA treatment) and timesaving compared to DNA extraction. However, PMA treatment was necessary for the quantification of viable VBNC-state cells, and PBS washing was required after treatment to analyze the ddPCR results. The reason for the analysis may be that the dye is generally fat-soluble and may penetrate the oil film. The negative and positive droplets were indistinguishable, so the result was negative. It was necessary to wash away the excess PMA dye. There was no significant difference between one and three washes, so one wash was recommended to avoid excessive washing. qPCR involves a continuous fluorescent signal analyzed with PCR time, while the PCR endpoint fluorescent signal is analyzed by ddPCR, and the endpoint signal was more sensitive to the background samples. Because heating and cooling cause sufficient DNA release, we also added 4°C for 10 min after ddPCR PCR at 95°C for 10 min and 37°C for 10 min for qPCR (qPCR minimum 37°C). Adding or not cooling the sample for 10 min did not affect the result, indicating that the DNA has been completely released after incubation at 95°C for 10 min.

This method has potential applications for the quantification of viable pathogens or other bacteria in samples that cannot be routinely cultured but still survive in the VBNC state. For example, it can be used to distinguish dead and VBNC bacteria in clinical or food samples in the risk assessment of V. cholerae or other pathogens in aquatic environments because common culture methods cannot detect the pathogen in the VBNC state. In summary, we established and evaluated a rapid and accurate quantitative PCR method for oil-enveloped bacteria to quantify VBNC V. cholerae cells. Treatment with PMA effectively eliminated the chromosomal DNA of dead cells. ddPCR combined with PMA treatment was better than qPCR for VBNC cell counts, and the lower limit of detection was improved. Although qPCR is less expensive and technically easier to perform, when absolute quantification of small numbers of target cells is performed, oil-enveloped bacterial ddPCR is a rapid method for quantification. Accurate quantification of the bacteria that survive in the VBNC state in the environment has an important impact on public health and food safety. Our method can be used for VBNC state research and the detection and quantification of VBNC state V. cholerae cells or other bacteria in samples from different environments, the clinic, and food.

## MATERIALS AND METHODS

### Bacterial strain, culture, and a mock sample of environmental water.

V. cholerae O1 El Tor strain C6706 was preserved in our laboratory. The experiment was completed in a biosafety level 2 (BSL-2) laboratory according to biosafety management regulations. The strains were stored in 20% (V/V) glycerol at −80°C and cultured on nutrient agar. Single colonies were suspended in Luria-Bertani (LB) medium (Oxoid, Basingstoke, UK) and incubated overnight with shaking (200 rpm) at 37°C. The cultures were then suspended in fresh LB broth (1:50, V/V) and shaken (200 rpm) at 37°C until mid-exponential-phase growth. V. cholerae VBNC cells were cultured in artificial seawater (ASW). ASW was washed twice and diluted to an optical density at 600 nm (OD_600_) = 1.0 (approximately 1 × 10^9^ CFU [CFU]/mL). Finally, the cultures were inoculated into ASW at a final concentration of 1 × 10^8^ CFU/mL. ASW was prepared from 40 g of sea salt (40 g/liter; Sigma-Aldrich, Inc., St. Louis, MO, USA) in distilled water and sterilized using 0.22-μm membrane filters (Millipore, USA). The exponential-phase cells were stored in ASW at 4°C with restricted oxygen. Culturable cells were counted by dilution in ASW every 5 days until no culturable cells could be detected from 1 mL of stock solution, which was considered evidence of entry into VBNC induction.

To test the count and lower limit detection of V. cholerae VBNC state cells in aquatic samples, three pond water samples were randomly collected and added to VBNC cells (laboratory cultured V. cholerae VBNC state cells at day 40). These water samples were negative for the V. cholerae *thyA* single-copy gene by PCR. The laboratory cultured VBNC cells were subjected to gradient dilution, and each dilution was mixed with aquatic pond water samples (1:1, V/V). The pond water samples mixed with ASW (1:1, V/V) were used as the negative control.

### PMA treatment.

In our previous study (19), viable cells were enumerated using PMA (Biotium, USA) combined with qPCR or ddPCR. Briefly, 200-μL aliquots of cells were treated with 20 μM PMA for 20 min in the dark and then exposed to light on ice for 15 min using a 650-W double-ended halogen lamp. PMA-treated samples were eluted once and thrice with phosphate-buffered saline (PBS), and noneluted samples were used as controls.

### DNA extraction.

Genomic DNA was extracted from 200-μL cell suspensions treated or not treated with PMA with a metal bath and a DNA extraction kit. In the metal bath, the samples were kept at 100°C for 10 min and then placed in an ice bath for 10 min and centrifuged at 2152 × *g* for 10 min. The DNA extraction kit method was performed according to the manufacturer’s instructions for the Wizard Genomic DNA Purification kit (Promega, Madison, WI, USA). Purified DNA was quantified using a NanoDrop 2000c spectrophotometer (Thermo Scientific, Asheville, NC, USA). DNA samples were stored at −20°C.

### Quantitative real-time PCR (qPCR).

The mixture for qPCR amplification contained 1 μL of template DNA, Premix *Ex Taq* (TaKaRa, Dalian, China), a 0.25 μM concentration of each forward (5′-ACA-3′) and reverse primer (5′-ATA-3′) targeting the single-copy gene *thyA*, and ultrapure water to a final volume of 20 μL. qPCR was performed in a LightCycler 96 system (Roche, Indianapolis, IN, USA). All qPCRs were performed in triplicate. The thermal cycling conditions were 10 min at 95°C, followed by 40 cycles of 5 s at 95°C and 30 s at 54°C, and then melt curve analysis from 65°C to 95°C with increments of 0.5°C for 5 s each. The enumeration results were considered negative if the Cq value was >35.

To prepare standard curves, DNA was extracted from purified V. cholerae O1 El Tor strain C6706, and standard curves were made by serially 10-fold diluting this DNA.

### Droplet digital PCR (ddPCR).

ddPCR was based on EvaGreen chemistry, and data analysis was performed via the Poisson calculation based on the fluorescence signal of the PCR endpoint. EvaGreen Supermix (2×) was purchased from Bio-Rad Laboratories. PCR was performed in a 20-μL volume containing 10 mL 2× EvaGreen Supermix, 1 mL DNA, 0.2 μM each primer, and 8.8 μL ddH_2_O. A Bio-Rad Automated Droplet Generator was used to generate droplets. Thermal cycling was performed using a Bio-Rad C1000 Touch™ Thermal Cycler with the following conditions: 95°C for 10 min; 40 cycles of 30 s at 95°C and 30 s at 54°C; and a final incubation at 4°C for 5 min and 90°C for 5 min. The oil-enveloped bacterial method released DNA in the first cycle condition (95°C for 10 min). After the reaction, droplets were analyzed using a QX200™ Droplet reader. Data analysis was performed using Bio-Rad QuantaSoft™ software.

In addition, the correspondence of CFU (CFU), qPCR, and ddPCR counting methods were also estimated. V. cholerae strain was diluted to OD_600_ = 0.02 (approximately 5 × 10^2^ CFU/mL) and cultured. CFU counting, PMA-qPCR, and PMA-ddPCR were performed per hour during the culture.

### Live/dead staining.

Live/dead staining was performed as described previously to examine VBNC viability (19). The cells were stained with a 0.6 μL mixture of SYTO9 and propidium iodide (1:1 v:v; Molecular Probes, Eugene, OR, USA) per 200 μL of suspension. After incubation in the dark for 15 min at 25°C, the stained cells were mounted on a glass slide. The cells were then examined with a Nikon ECLIPES 80i microscope. Images were captured with NIS-Elements F3.2 microscopy software (Nikon).

### Statistical analysis.

Figures were generated from three replicate values using GraphPad Prism software. Each replicate value was the mean value from a triplicate measurement. All statistical analyses were performed using GraphPad Prism. Group means and standard deviations (SDs) were analyzed by Student's *t* test. For all tests, the difference was considered statistically significant if the *P* value is less than 0.05. The VBNC rate is the percentage of VBNC state bacteria over total bacteria.
