# Supplementary material for: Absolute Quantification of Viable but Nonculturable Vibrio cholerae Using Droplet Digital PCR with Oil-Enveloped Bacterial Cells
Source: Microbiol Spectr. 2022 Jun 28;10(4):e00704-22. doi: 10.1128/spectrum.00704-22 (PMC9430983; doi:10.1128/spectrum.00704-22)

Supplemental file: Figure S1. Correspondence of cell counting among CFU counting, PMA-qPCR and PMA-ddPCR methods. *thyA* gene copy counting with PMA-qPCR and PMA-ddPCR was listed as the example.

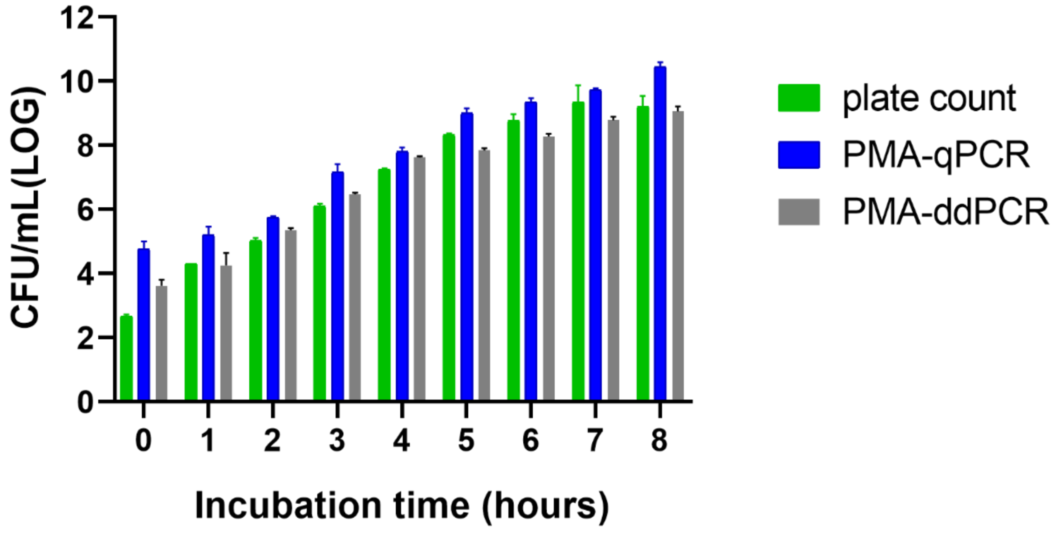

Supplement: Supplemental file 1 — Fig. S1. Download spectrum.00704-22-s0001.pdf, PDF file, 0.05 MB [file spectrum.00704-22-s0001.pdf]
